# Complications After Primary Breast Augmentation: Capsular Contracture

**Published:** 2015-07-20

**Authors:** Karan Chopra, Arvind Gowda, Luther H. Holton, Sheri Slezak, Devinder P. Singh

**Affiliations:** ^a^Division of Plastic Surgery, University of Maryland School of Medicine, Baltimore, MD; ^b^Department of Plastic Surgery, The Johns Hopkins University School of Medicine, Baltimore, MD; ^c^Eastern Virginia Medical School, Norfolk, VA

**Keywords:** revisionary breast augmentation, complications, capsular contracture, neopectoral, biofilm

**Figure:**
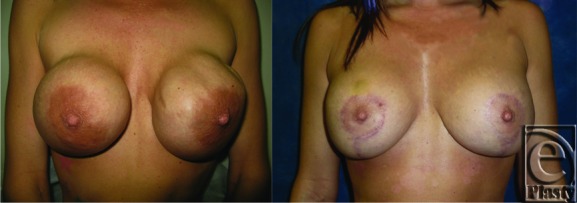


## DESCRIPTION

Capsular contracture is a common complication following breast augmentation. Although the etiology is unknown, it is hypothesized that subclinical infection contributes to the process. Surgical treatment strategies must take this into account to avoid recurrence.

## QUESTIONS

1. **What are the most common complications after breast augmentation?**
2. **What is capsular contracture?**
3. **What is the role of biofilm in capsular contracture?**
4. **What are the treatments of capsular contracture?**

## DISCUSSION

The most common complications following breast augmentation are capsular contracture, asymmetry, implant malposition, hematoma, and infection.^1^^,^^2^ Capsular contracture is the most common reason for reoperation within 4 years of primary augmentation.^1^

All surgically implanted foreign bodies become encapsulated over time. Following breast augmentation, an abnormally thick or contracted capsule may form due to interactions between inflammatory cells, fibroblasts, and extracellular matrix constituents.^3^ When this becomes clinically apparent, it is termed “capsular contracture.” The Baker classification grades capsular contracture on the basis of breast firmness, palpability of the implant, distortion of the breast or visibility of the implant, and breast tenderness.

Infectious etiologies are theorized to play a role in capsular contracture. Burkhardt et al^4^ proposed the idea that subclinical infection contributes to this processes in 1981 after finding a 71% positive culture rate in open capsulotomy specimens. *Staphylococcus epidermidis* was implicated in 87% of positive cultures. The pathogenicity of *S epidermidis* results from the organism's ability to produce a biofilm.^5^ Biofilms comprise bacterial colonies that secrete extracellular polymeric substances composed mainly of polysaccharide and protein constituents. These secretions form an insulating layer around bacterial colonies, protecting them from host defense and antimicrobial agents.^5^ Surgically implanted and indwelling foreign bodies are particularly susceptible to biofilm formation. In a 2010 study utilizing a porcine model of breast augmentation, Tamboto et al^5^ found that implant pockets inoculated with *S epidermidis* were strongly associated with biofilm formation compared with controls (*P* = .0095). In addition, authors demonstrated that biofilm formation was associated with a 4-fold increased risk of capsular contracture.^5^

Treatment of capsular contracture typically requires operative intervention. Surgeons initially addressed this with closed or open capsulotomy alone. This was abandoned because of high recurrence rates and an understanding that the implant pocket must be altered in some way. At a minimum, partial capsulectomy should be performed. Total capsulectomy provides a better long-term result but may be difficult to safely achieve.^6^ Additional treatment options include changing the location of the implant and use of acellular dermal matrix. Changing the location of the implant involves creation of a new pocket free from bacterial contamination. Patients with subglandular implants may have them replaced with subpectoral implants.^7^ For patients with subpectoral implants, it may be unfeasible to switch to a subglandular position due to overlying soft-tissue atrophy from the pressure of the previous implant. Instead, a neosubpectoral pocket can be created superficial to the capsule following obliteration of the capsular space.^7^ Acellular dermal matrix is a processed human or porcine dermis with removed cellular elements and dermal appendages. This material becomes incorporated into host tissue through revascularization and protein turnover.^1^ There is a growing body of evidence suggesting a decreased recurrence of capsular contracture when acellular dermal matrix is used in implant revision.^1^ Histological assessment has shown development of significantly less collagen and myofibroblast proliferation in regions of acellular dermal matrix—covered implant compared with the surrounding capsule. In fact, a sharp demarcation at the interface between acellular dermal matrix and the surrounding capsule can be visualized under the microscope.^7^^,^^8^ Nonsurgical treatments of capsular contracture such as leukotriene receptor antagonists and steroids do exist, but the evidence supporting their use is weak. In addition, both have been associated with adverse effects.

Current treatment strategies for capsular contracture acknowledge the likely role subclinical infection and biofilm formation play in this process. Capsulectomy, implant pocket change, and use of acellular dermal matrix can help prevent the recurrence of capsular contracture.
